# Supplementary material for: Acid sphingomyelinase is a gatekeeper of placental labyrinthine architecture and function
Source: Development. 2025 Oct 6;152(19):dev204425. doi: 10.1242/dev.204425 (PMC12539202; doi:10.1242/dev.204425)
Supplement: Supplementary information [file develop-152-204425-s1.pdf]

Development: doi:10.1242/dev.204425: Supplementary information

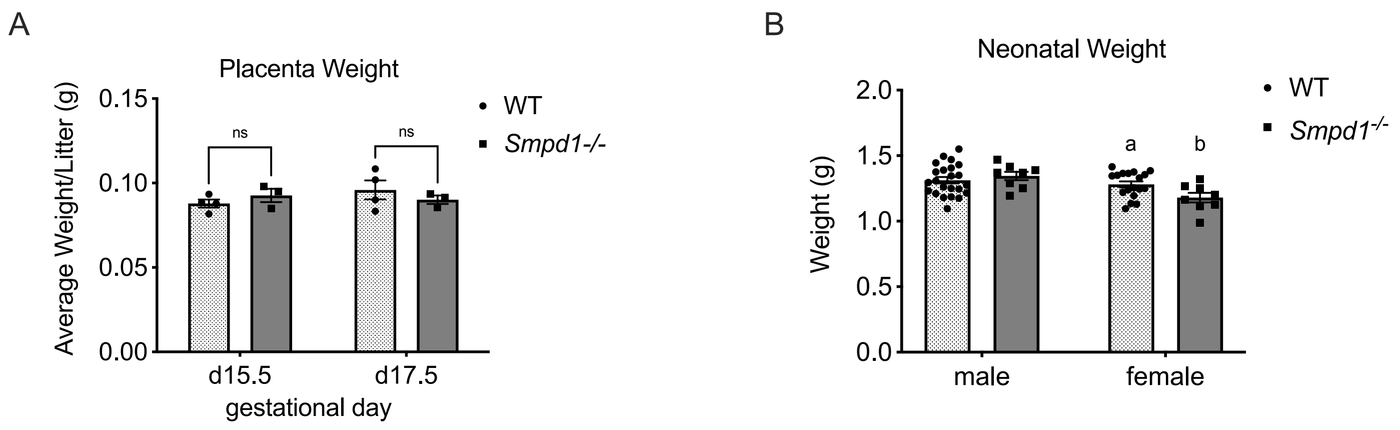

Fig. S1. Placenta and neonatal weights of *Smpd1-/-* mice

(a) Average placental weight per litter from wildtype and *Smpd1-/-* mice at gestational days d15.5 and d17.5 (Two-way ANOVA) (b) Male and female neonatal weights are similar between WT and *Smpd1-/-* (Two-way ANOVA). All data is shown as mean ± SEM and different letters represent statistical significance ($p<0.05$).

Development • Supplementary information

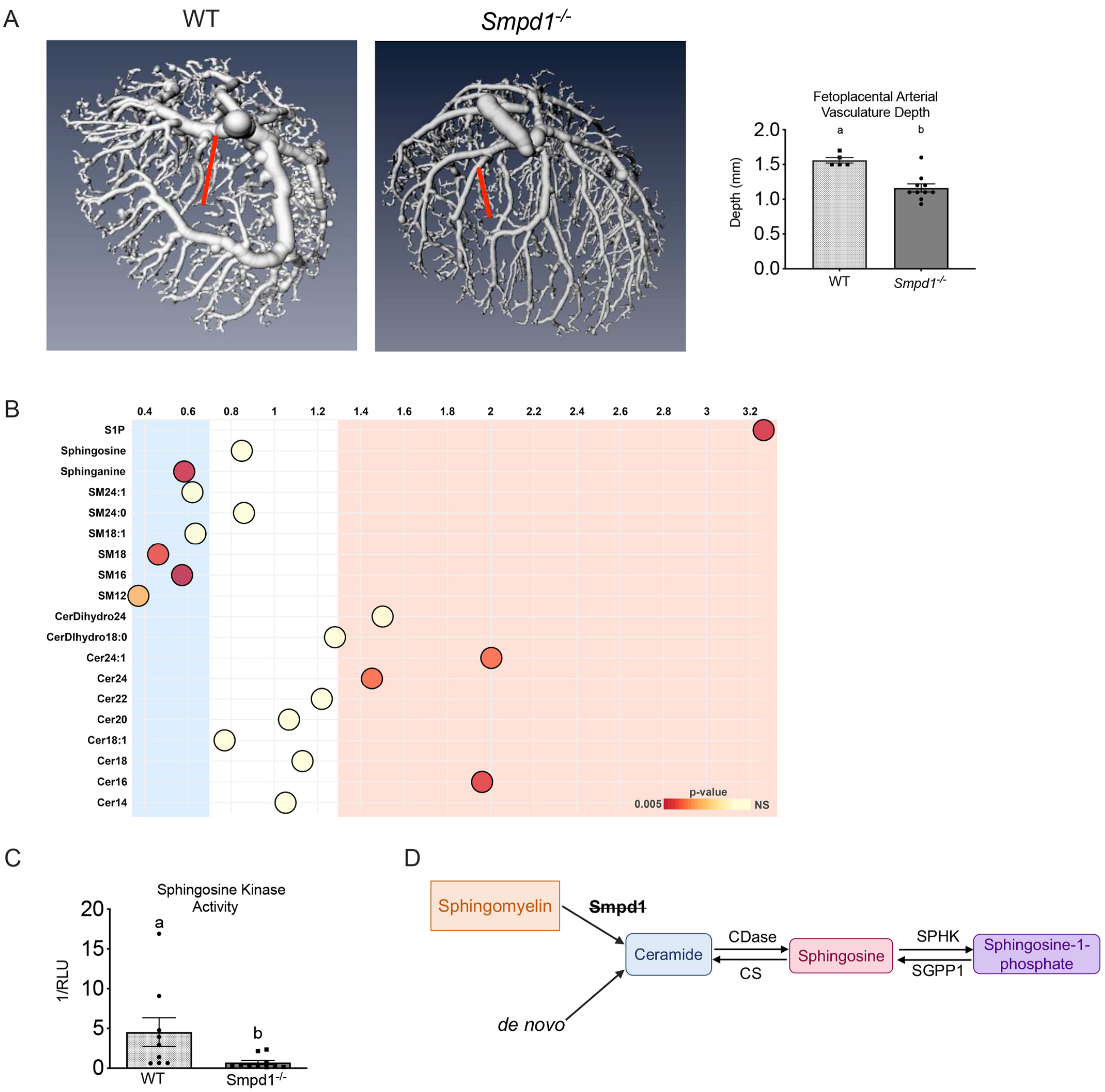

Fig. S2. Placental vasculature depth and sphingolipid metablism in *Smpd1$^{-/-}$* placentas. (a) Fetoplacental arterial vasculature was reconstructed and visualized using Amira^{TM} in lower panel from WT and *Smpd1$^{-/-}$* of the placental vasculature cast for the quantitation of the depth (red lines) of the vasculature tree. (b) Dot plot representation of mean ratio of individual sphingolipid metabolites obtained from LC MS/MS analysis of 3 WT and 3 *Smpd1$^{-/-}$* placentas. Metabolites decreased in mutants are within the orange field and those increased are in blue field. Values shown are a ratio of fold change. Intensity of dot represents significance. (c) Sphingosine kinase (SPHK) activity was significantly reduced in *Smpd1$^{-/-}$* /placentas. (d) Schematic of sphingolipid metabolic pathway. Sphingolipid synthesis is dependent on the production of ceramide either *de novo* or from the hydrolysis of sphingomyelin by Smpd1(sphingomyelin phosphodiesterase 1). Ceramide is broken down into sphingosine by ceramidase (CDase), while this is a reversible reaction by Ceramide Synthase (CS). Sphingosine Kinase (SPHK) phosphorylates sphingosine to convert it to sphingosine-1-phosphate (S1P), that can be reversed by SGPP1 (Sphingosine-1-phosphate phosphatase 1). All data is shown as mean ± SEM, and different letters represent statistical significance ($p<0.05$).

Development • Supplementary information

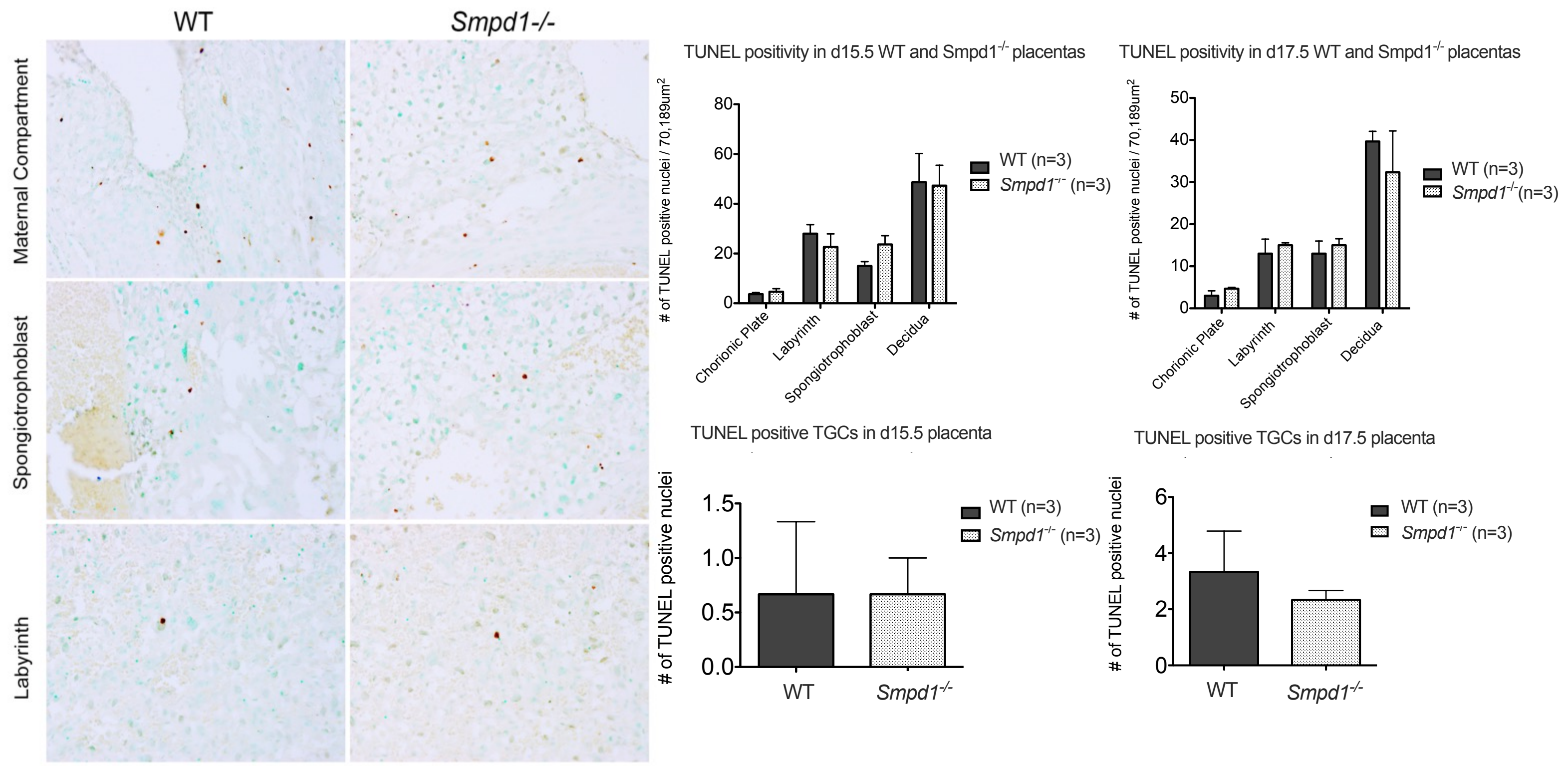

Fig. S3. Apoptotic cell death is not altered in *Smpd1*$^{-/-}$ placentas. Representative images of d15.5 WT and *Smpd1*$^{-/-}$ placentas labelled with TUNEL at 20x. There is no significant difference in the number of TUNEL positive nuclei between d15.5 WT and *Smpd1*$^{-/-}$ placentas and no significant difference in the total number of TUNEL positive TGC.

Development • Supplementary information
